# Exploration of virulence and immune evasion functions of the candidate vaccine antigen SpyAD in the globally disseminated M1T1 group A *Streptococcus* strain

**DOI:** 10.1128/mbio.00683-25

**Published:** 2025-06-11

**Authors:** Alexandra Stream, Samira Dahesh, Lamar Thomas, Nina J. Gao, Elisabet Bjånes, Kalisa Kang, Truman Koh, Neeraj Kapoor, Victor Nizet

**Affiliations:** 1 Medical Scientist Training Program, University of California, San Diego School of Medicine 12220 , La Jolla, California, USA; 2 Division of Host-Microbe Systems and Therapeutics, Department of Pediatrics, University of California, San Diego School of Medicine 12220, La Jolla, California, USA; 3 Vaxcyte, Inc. 681082 , San Carlos, California, USA; 4 Skaggs School of Pharmacy and Pharmaceutical Sciences, University of California, San Diego School of Medicine 12220, La Jolla, California, USA; Harvard Medical School, Boston, Massachusetts, USA

**Keywords:** *Streptococcus pyogenes*, vaccine antigen, virulence factors, immune evasion

## Abstract

Group A *Streptococcus* (GAS; *S. pyogenes*) is a significant human pathogen responsible for a wide range of diseases, including invasive conditions with high mortality rates, for which no FDA-approved vaccine exists. The membrane-bound protein *S. pyogenes* adhesion and division protein (SpyAD) is highly conserved among all sequenced GAS isolates and has emerged as a promising vaccine antigen, but its precise role in GAS pathogenesis remains unclear. Here, we investigate the contribution of SpyAD in establishing and maintaining infection in M1T1 GAS, a persistent and globally disseminated strain that is the most frequently isolated serotype in both invasive and noninvasive infections. We found that SpyAD is critical for GAS invasion of immortalized human pharyngeal and vaginal epithelial cells, as loss of SpyAD significantly impaired bacterial invasion. SpyAD deficiency also resulted in impaired biofilm formation. These features suggest that SpyAD facilitates bacterial persistence and contributes to GAS virulence. In addition, SpyAD enhanced GAS resistance to neutrophil-mediated killing, potentially through phagocytosis-dependent mechanisms. *In vivo* experiments demonstrated that SpyAD deficiency leads to decreased bacterial burden and prolonged survival in a murine model of systemic infection, as well as reduced colonization of the murine vaginal mucosa, further highlighting its role in GAS virulence. These findings underscore the importance of SpyAD as a factor in GAS pathogenesis in the M1T1 strain and support its potential as a target for vaccine development.

**IMPORTANCE:**

Group A *Streptococcus* (GAS) infections remain prevalent worldwide, with recent surges in severe invasive cases causing high morbidity and mortality. To effectively combat these invasive infections, a more thorough understanding of GAS pathogenesis—particularly mechanisms of immune invasion and evasion—is critical. In this study, we address this need by elucidating the role of the candidate GAS vaccine antigen SpyAD in these key processes in the highly prevalent M1T1 GAS strain. SpyAD enables GAS to evade neutrophil killing and facilitates epithelial cell invasion, likely helping the bacterium circumvent immune defenses. Moreover, SpyAD is important for systemic infection and vaginal mucosal infection *in vivo*. Vaccination targeting SpyAD may generate opsonophagocytic antibodies to improve GAS clearance and fortify protection against invasive disease.

## INTRODUCTION

Group A *Streptococcus* (GAS; *S. pyogenes*) is a major human pathogen responsible for a broad spectrum of diseases, ranging from pharyngitis to severe invasive conditions such as necrotizing fasciitis and streptococcal toxic shock syndrome. GAS ranks among the top 10 infectious agents causing human mortality, with approximately 500,000 deaths globally each year attributed to GAS infections (1, 2). Invasive GAS (iGAS) infections are particularly concerning due to their increasing incidence worldwide and the ability of invasive strains to persist despite prolonged antibiotic exposure, even without genetic resistance (2–6). This underscores the need for improved prevention and treatment strategies. Uncovering GAS pathogenesis, particularly in invasive disease contexts, is critical for these efforts.

Central to iGAS pathogenesis is the ability of the bacterium to evade the innate immune response and spread to deeper tissues. GAS entry and survival in epithelial cells are thought to facilitate breach of physical barriers, persistence within the host, and penetration of deeper tissues (7). Such intracellular GAS may explain bacterial persistence despite appropriate antibiotic therapy and contribute to recurrent tonsillitis (8–10). Notably, GAS strains derived from patients with bacterial eradication failure showed improved survival within epithelial cells *in vitro* (11). Enhanced epithelial cell invasion may also enable GAS to evade host immune responses (12, 13). Resistance to neutrophil-mediated killing is a particularly prominent virulence attribute of GAS, involving mechanisms such as inhibition of phagocytosis, suppression of oxidative burst, and resistance to antimicrobial peptides (14). These strategies are critical for the pathogen’s survival in the bloodstream and other tissues; thus, the identification of factors involved in these processes is important for developing new therapeutic interventions.

The GAS membrane-bound protein, SpyAD (Spy0269), has emerged as a promising candidate for inclusion in a GAS vaccine formulation and is ubiquitous across GAS clinical strains (15–18). SpyAD is highly conserved, with at least 98% amino acid sequence identity in all published genomes (16). Furthermore, its surface localization makes SpyAD an easily accessible target for the immune system, reinforcing its potential as a component of a GAS vaccine. Subcutaneous SpyAD immunization protected mice against intranasal or intravascular GAS infection (16), and it has been included in additional multivalent GAS subunit vaccines protective against mucosal or systemic infection (17). Our previous work has found that SpyAD could serve as a carrier protein to conjugate to the universally conserved group A cell wall carbohydrate (GAC), as part of a multivalent vaccine (VAX-A1) formulation (VaxCyte, Inc.) that protects mice against systemic infection (15). Although anti-SpyAD antibodies can enhance opsonophagocytic clearance of GAS *in vitro* (15), the specific roles of SpyAD in promoting GAS pathogenesis remain incompletely understood. Prior research has suggested that SpyAD can mediate GAS adhesion to host epithelial cells and participate in bacterial division processes, as indicated by its colocalization with the bacterial septum and division protein FtsZ and its influence on bacterial chain length (19).

While SpyAD shows potential as a vaccine antigen, its specific contributions to GAS pathogenesis are not fully elucidated. In this study, we utilize a ∆*spyAD* mutant in the M1T1 GAS strain to clarify the role of SpyAD in bacterial pathogenesis by investigating its involvement in epithelial cell interactions and resistance to innate immune responses and by assessing its virulence function using murine models of infection. We have selected the M1T1 strain for this study because it is the most prevalent GAS serotype associated with pharyngeal, skin, and invasive infections (20–22). Since the mid-1980s, M1T1 has been the predominant strain driving the resurgence of severe streptococcal infections, including sepsis, toxic shock-like syndrome, and soft tissue invasion (23). Its persistence and virulence have been largely attributed to the acquisition of phage-encoded factors such as the superantigen SpeA and the nuclease Sda1, which promotes immune evasion by degrading neutrophil extracellular traps (20, 24, 25). In addition, M1T1 GAS acquired a 36 kb chromosomal region from M12 GAS that drives enhanced expression of the extracellular toxins NAD+-glycohydrolase (NADase) and streptolysin O (SLO), further increasing its virulence (26, 27). Given its clinical relevance, M1T1 provides an optimal background for dissecting the functional role of SpyAD in GAS pathogenesis. By studying SpyAD in the M1T1 GAS strain, we aim to provide deeper insights into the multifaceted role of SpyAD in virulence and its desirability as a target for vaccine development.

## RESULTS

### SpyAD immunization provides protection in a murine skin infection model and boosts GAS killing in human whole blood

**Fig 1:**
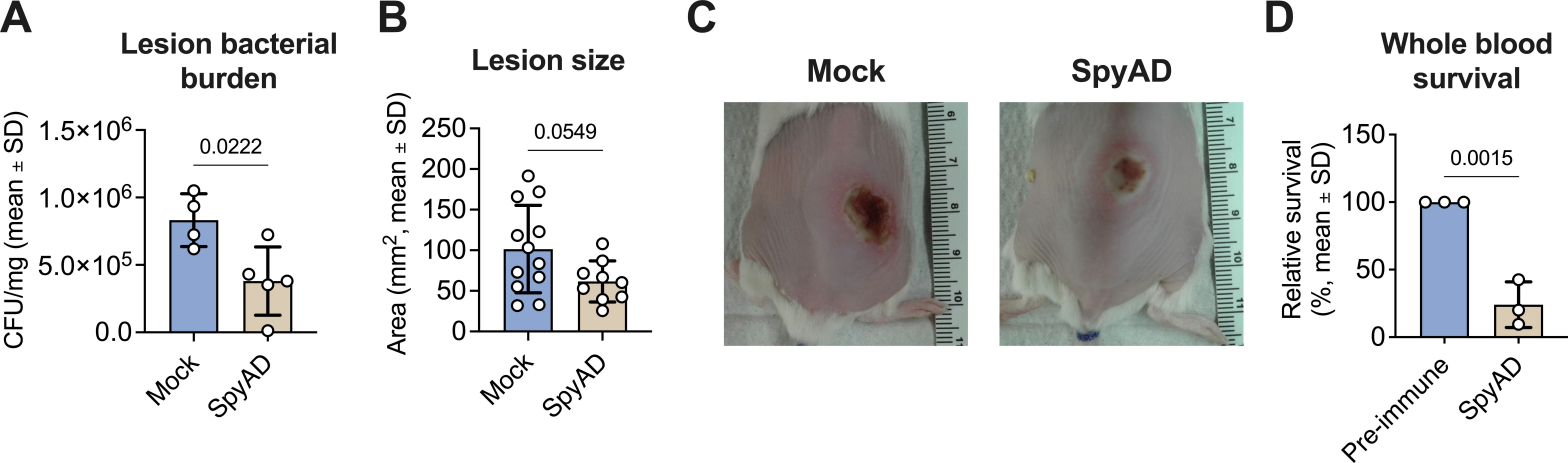
SpyAD immunization has protective effects against GAS *in vivo* and *in vitro*. (**A**) Bacterial burden from immunized female CD-1 mice infected intradermally with 10^6^ CFU GAS M1 89155. Mice were immunized on days 0, 14, and 28 with recombinant SpyAD adjuvanted with alum or a “mock” condition (PBS and alum), followed by skin infection 14 days past the final vaccination. Bacterial CFUs were counted from homogenized skin samples at 3 days post-infection. Each data point represents one mouse/biological replicate. Data are analyzed by Student’s *t* test and have an effect size equal to 0.5499 as calculated by eta squared (η^2^). (**B**) Corresponding lesion area (mm^2^) measured at 3 days post-infection. Each data point represents one mouse/biological replicate. Data are analyzed by Student’s *t* test (η^2^ = 0.1805). (**C**) Representative images of skin lesions on day 3 post-infection are shown. (**D**) Survival of wild-type M1T1 strain 5448 GAS opsonized with SpyAD rabbit antisera compared to pre-immune antisera in whole human blood for 1 h. Data are pooled from three independent experiments with different human blood donors, with each data point representing the mean of three technical replicates per experiment. Data are analyzed by Student’s *t* test (η^2^ = 0.9382). Bar graphs in panels A, B, and D show the mean with error bars representing standard deviation (SD). *P*-values are displayed on each graph above the corresponding comparison.

Previous studies have shown that SpyAD is immunogenic in rabbits and mice (15–17). To further examine the protective effects of SpyAD immunization, we vaccinated female wild-type CD-1 mice intramuscularly at day 0 (prime), then on days 14 and 28 (boosters), with SpyAD plus Alhydrogel 2% aluminum hydroxide adjuvant (alum) as an adjuvant. Phosphate-buffered saline (PBS) with alum alone was used as a mock control. Fourteen days after the final vaccination, mice were challenged intradermally with 10^6^ colony-forming units (CFU) of the invasive GAS M1 serotype strain 89155. At 3 days post-infection, the skin lesions were quantified for area size and harvested for recovery of GAS bacterial burden. The bacterial load (CFU/gram) in the skin was significantly lower in SpyAD-immunized mice compared to mock-immunized controls (Fig. 1A). Although not statistically significant, a trend of reduced lesion size from SpyAD-immunized mouse lesions was observed compared to the mock-immunized mouse lesions (Fig. 1B and C), consistent with previous findings using the VAX-A1 formulation, which includes SpyAD as part of the multivalent components (15). To examine SpyAD’s role in enhancing opsonophagocytic killing, we tested the effect of SpyAD rabbit antisera (15) on GAS survival in whole heparinized human blood. Wild-type M1T1 serotype strain 5448 GAS opsonized with SpyAD antisera showed significantly reduced survival compared to those treated with pre-immune sera (Fig. 1D). These data further support the protective role of anti-SpyAD antibodies in enhancing opsonophagocytic killing by immune cells in human blood.

### Loss of SpyAD in M1T1 GAS increases bacterial chain length without affecting growth or capsule production

**Fig 2:**
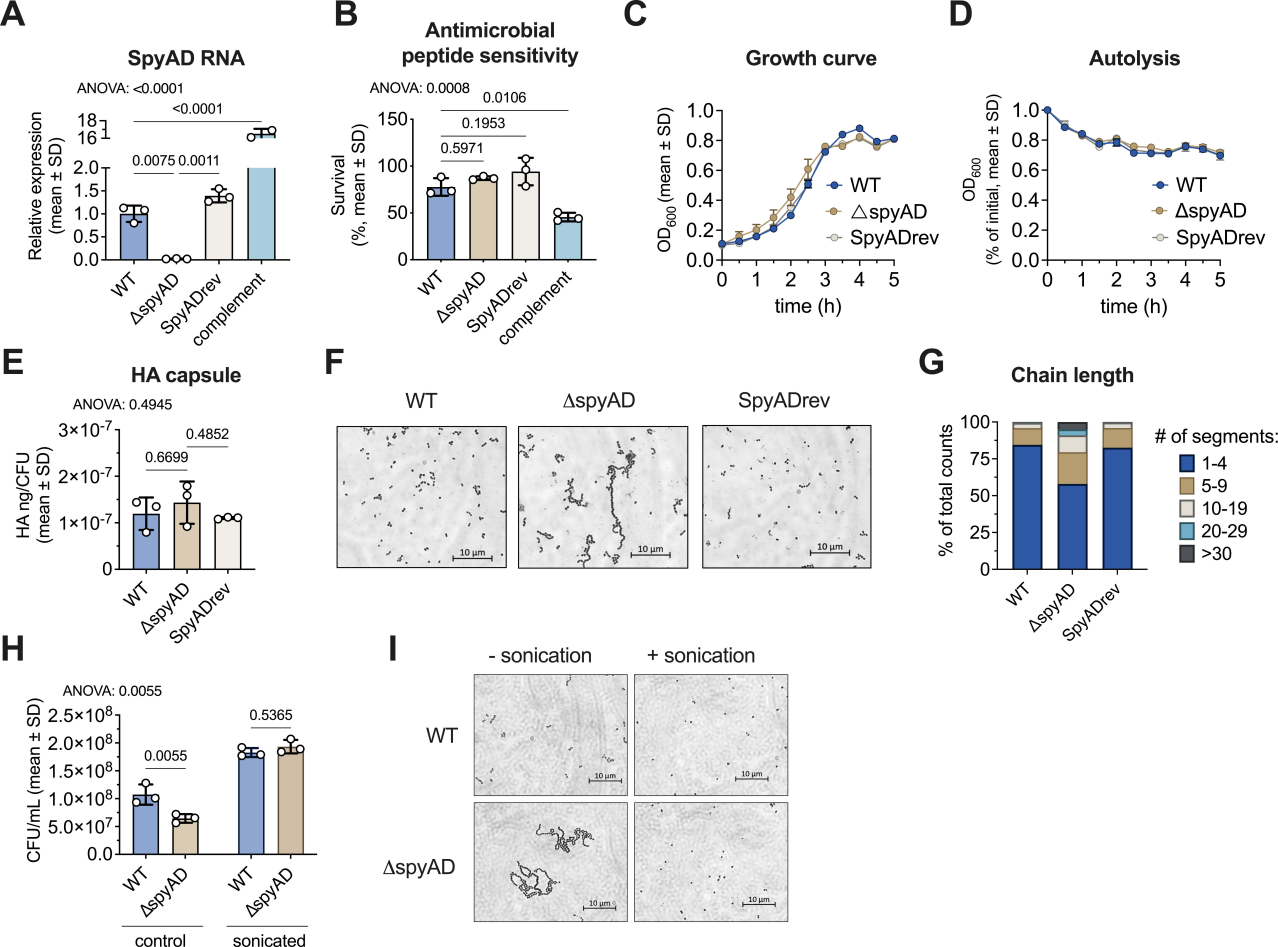
Δ*spyAD* mutant generation in M1T1 GAS and characterization. (**A**) qRT-PCR analysis of SpyAD mRNA expression in logarithmic phase bacteria, normalized to GAS gene, *gyrA*. Data are analyzed by one-way ANOVA with Tukey’s multiple posttest (η^2^ = 0.999). (**B**) Survival was calculated as the percent of inoculum with 8 µM antimicrobial peptide, LL-37 cathelicidin, after 30 min incubation. Data are analyzed by one-way ANOVA with Tukey’s multiple posttest (η^2^ = 0.8633). (**C**) OD_600_-based growth in THB medium, measured every 30 min for 5 h. (**D**) OD_600_-based survival after induction of autolysis by washing cells with ice-cold H_2_O followed by incubation in 0.05% Triton X-100, with optical density measured every 30 min for 5 h. (**E**) Hyaluronic acid capsule levels determined by Hyaluronan Quantikine ELISA kit (R&D Systems). Data are pooled from three independent experiments, with each data point representing the mean of two technical replicates per experiment. Data are analyzed by one-way ANOVA with Tukey’s multiple posttest (η^2^ = 0.2092). (**F**) Representative images of gram-stained WT, ∆*spyAD*, and SpyADrev GAS strains. (**G**) Corresponding quantification of chain length of each strain calculated as percent of total counts. (**H**) CFU/mL of wild-type or ∆*spyAD* GAS with or without sonication (2 × 3 s) of OD_600_ = 0.4 liquid culture. Data are pooled from three independent experiments performed in triplicate, with each dot representing the mean of the three technical replicates. Data are analyzed by two-way ANOVA with Tukey’s multiple posttest (interaction η^2^ = 0.0625; sonication factor η^2^ = 0.8830; strain factor η^2^ = 0.02196). (**I**) Representative images of wild-type or ∆*spyAD* GAS before and after sonication (2 × 3 s) to break chains into individual cocci. Panels **A**–**D**, **F**, **G**, and **I** show one representative graph or image set from three independent experiments. All graphs show mean ± SD, and *P*-values are shown above each corresponding comparison. *P*-values of the overall ANOVA test are displayed on the top left corner of the graph when applicable.

Given the protective effects of SpyAD immunization, we explored its virulence roles in more detail using a plasmid integrational knockout strain (Δ*spyAD*) constructed in the well-characterized invasive GAS M1T1 serotype strain 5448 (15). The Δ*spyAD* mutant has previously shown reduced surface binding to SpyAD rabbit antisera compared to the wild-type parent strain (15). mRNA analysis verified the absence of *spyAD* expression in the Δ*spyAD* mutant (Fig. 2A). Complementation of the mutant phenotype was first attempted by cloning *spyAD* on a multicopy expression plasmid (pDC123) and transforming this construct into the mutant (Δ*spyAD* +pDC-SpyAD); however, the plasmid-complemented strain had markedly increased SpyAD RNA expression compared to wild-type (Fig. 2A) and evidence of cell wall integrity changes reflected in increased susceptibility to the cationic cell-wall active antimicrobial peptide LL-37 compared to both wild-type and Δ*spyAD* GAS strains (Fig. 2B). To circumvent misinterpretation of virulence phenotypes related to toxicity from SpyAD overexpression in the plasmid-complemented strain, a revertant strain was generated by serial passage of the Δ*spyAD* strain in the absence of antibiotic selection. RNA expression of SpyAD in the revertant was restored to similar levels of wild-type GAS (Fig. 2A), and this strain (SpyADrev) was used as a control in subsequent experiments to exclude polar effects of the original mutation.

Growth in liquid culture, measured by optical density at 600 nm (OD_600_), was unaffected by SpyAD deletion (Fig. 2C), and there was no increase in autolysis (Fig. 2D). In addition, hyaluronic acid capsule levels, an important GAS virulence factor (28, 29), were equal among strains (Fig. 2E). Consistent with prior literature (19), the loss of SpyAD increased bacterial chain length compared to wild-type and SpyADrev (Fig. 2F and G). To ensure accurate CFU enumeration in subsequent experiments, probe sonication was used to break bacterial chains of the wild type and ∆*spyAD* mutant, which normalized CFU counts between the two strains (Fig. 2H and I).

### SpyAD facilitates epithelial cell interactions and promotes biofilm formation in M1T1 GAS

**Fig 3:**
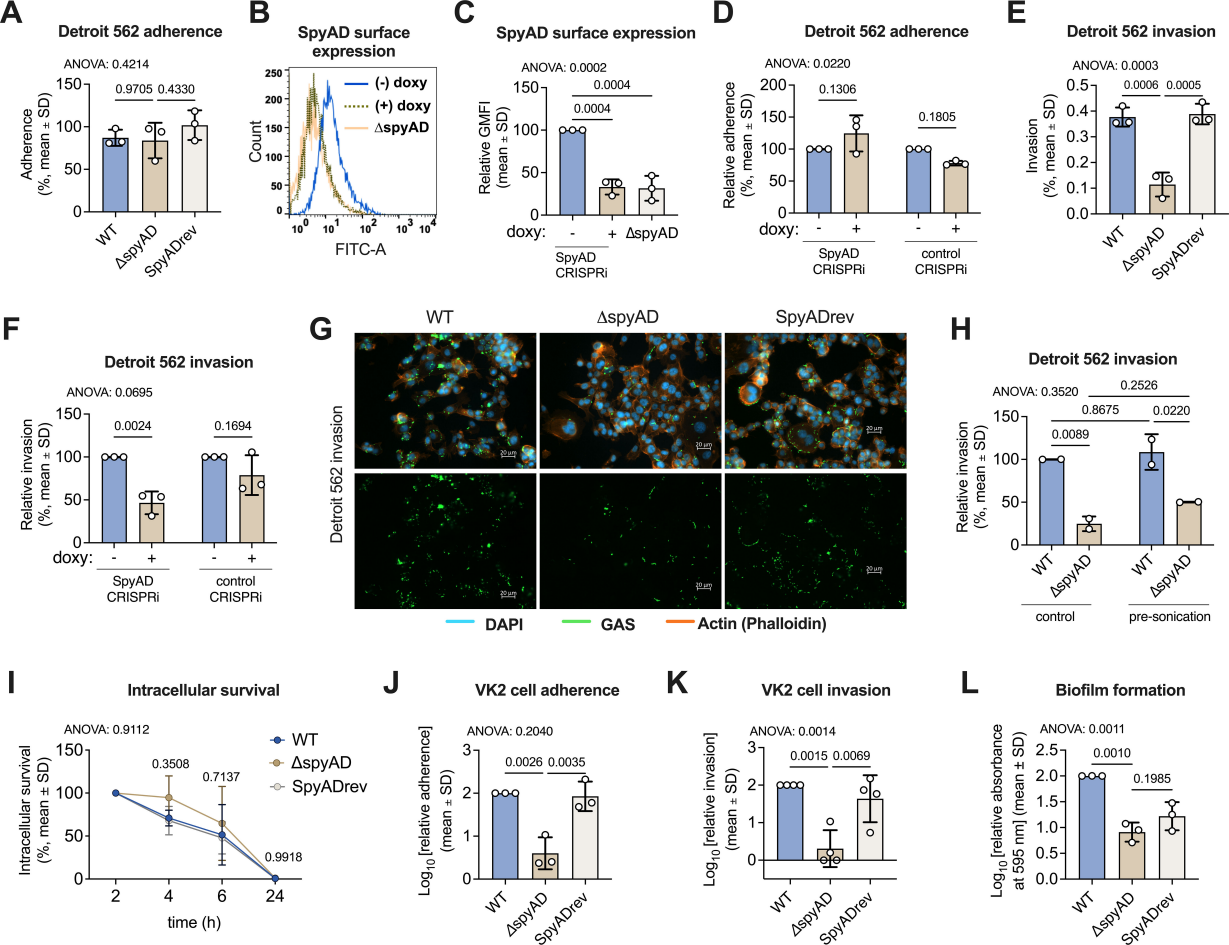
SpyAD contributes to epithelial cell invasion, vaginal cell adherence, and biofilm formation in M1T1 GAS. (**A**) GAS adherence to Detroit 562 pharyngeal epithelial cells at MOI = 5, calculated as a percent of initial inoculum, after 30 min incubation. Data show three independent experiments done in triplicate, with each point showing the mean of the three technical replicates. Data are analyzed by one-way ANOVA with Tukey’s multiple posttest (η^2^ = 0.2503). (**B**) Representative flow cytometry histogram and (**C**) geometric mean fluorescent intensity measures from three independent experiments of SpyAD surface expression of CRISPRi strains in the presence or absence of 20 ng/mL doxycycline compared to ∆*spyAD* mutant strain. Data are pooled from three independent experiments, all normalized to CRISPRi “minus doxycycline” control. Data are analyzed by one-way ANOVA with Tukey’s multiple posttest (η^2^ = 0.9396). (**D**) Detroit 562 adherence (MOI = 5, 30 min) of CRISPRi strains grown in the presence or absence of 20 ng/mL doxycycline. Adherence is calculated and graphed relative to the “minus doxycycline” condition for each strain. Data show combined three independent experiments done in triplicate, with each point showing the mean of the three technical replicates. Data are analyzed by two-way ANOVA with Tukey’s multiple posttest (interaction η^2^ = 0.3334; strain factor η^2^ = 0.3334; ±doxy factor η^2^ = 0.0009870. (**E**) GAS invasion of Detroit pharyngeal epithelial cells at MOI = 5, calculated as a percent of initial inoculum after 2 h of incubation and 2 h with penicillin/gentamicin to kill extracellular bacteria. Data reflect combined three independent experiments done in triplicate, with each point showing the mean of the three technical replicates. Data are analyzed by one-way ANOVA with Tukey’s multiple posttest (η^2^ = 0.9331). (**F**) Detroit 562 invasion assay (MOI = 5, 2 h) of CRISPRi strains grown in the presence or absence of 20 ng/mL doxycycline. Relative invasion of input is calculated and normalized to “minus doxycycline” control for each strain. Data reflect three independent experiments done in triplicate, with each point showing the mean of the three technical replicates. Data are analyzed by two-way ANOVA with Tukey’s multiple posttest (interaction η^2^ = 0.1093; strain factor η^2^ = 0.1093; ±doxy factor η^2^ = 0.5822). (**G**) Microscopic fluorescent invasion images using Alexa fluor 647-phalloidin (far-red, actin), DAPI (blue, DNA), and GAS pre-stained with CellBrite Membrane Stain 488 (green). (**H**) GAS invasion of Detroit 562 cells using sonicated or control bacteria. “Pre-sonication” refers to the treatment used to break up bacterial chains prior to the invasion assay. Data show combined two independent experiments done in triplicate, with each point showing the mean of the three technical replicates. Data are analyzed by two-way ANOVA with Tukey’s multiple posttest (interaction η^2^ = 0.01379; ±pre-sonication factor η^2^ = 0.05656; strain factor η^2^ = 0.8798). (**I**) Intracellular survival of GAS (MOI = 5) in Detroit 562 cells, normalized to 2 h timepoint post-antibiotic addition to kill extracellular bacteria (invasion). Data show pooled three independent experiments performed in triplicate. *P*-values on the graph indicate a comparison between WT and ∆*spyAD* mutant GAS. Data are analyzed by two-way ANOVA with Tukey’s multiple posttest (interaction η^2^ = 0.01734; time factor η^2^ = 0.7821; strain factor η^2^ = 0.01550). (**J**) GAS adherence to VK2 vaginal epithelial cells for 30 min at MOI = 10, normalized to WT. Data reflect combined three independent experiments done in triplicate, with each point showing the mean of the three technical replicates. The Shapiro-Wilk normality test demonstrated a log-normal distribution of the data, so values were log_10_-transformed before graphing and analysis by one-way ANOVA with Tukey’s multiple posttest (η^2^ = 0.6331). (**K**) GAS invasion of VK2 vaginal epithelial cells for 2 h at MOI = 10, normalized to WT. Data reflect combined three independent experiments done in triplicate, with each point showing the mean of the three technical replicates. Data were log-normally distributed (Shapiro-Wilk assessment), so values were log_10_-transformed before graphing and analysis by one-way ANOVA with Tukey’s multiple posttest (η^2^ = 0.6484). (**L**) Biofilm formation was determined by crystal violet assay pooled from three independent experiments of 12 technical replicates each. Each data point represents the mean of 12 technical replicates. Data were log-normally distributed (Shapiro-Wilk assessment), so values were log_10_-transformed before graphing and analysis by one-way ANOVA with Tukey’s multiple posttest (η^2^ = 0.9834). All graphs show mean ± SD, and *P*-values are shown above each corresponding comparison. *P*-values of the overall ANOVA test are displayed on the top left corner of the graph when applicable.

M1T1 GAS can adhere to epithelial cells, invade them, and replicate intracellularly in the cytosol (30). Epithelial cell adherence and invasion allow GAS to establish infection and slow host immune clearance during pharyngitis (7, 13). To understand SpyAD’s role in these pathogenic mechanisms in M1T1 GAS, we assessed GAS adherence and invasion of cultured human Detroit 562 pharyngeal epithelial cells. Loss of SpyAD did not affect adherence to the epithelial cells (Fig. 3A), a finding we corroborated using a CRISPR interference (CRISPRi) system (31) to knock down SpyAD expression in wild-type GAS M1T1 5448, which showed no change in adherence after doxycycline-induced knockdown (Fig. 3B through D). However, the ∆*spyAD* mutant exhibited significantly reduced invasion of Detroit 562 cells compared to wild type and SpyADrev (Fig. 3E), an effect confirmed by CRISPRi SpyAD knockdown (Fig. 3F). Microscopy further revealed decreased intracellular ∆*spyAD* GAS compared to wild type (Fig. 3G). Of note, the longer chains observed in the ∆*spyAD* mutant did not play a significant role in its invasion phenotype, as the difference between WT and ∆*spyAD* GAS persisted even after both strains were sonicated prior to the assay to disrupt long chains (Fig. 3H). Furthermore, the observed reduction in intracellular recovery of ∆*spyAD* mutant bacteria was not attributable to impaired intracellular survival following uptake, as the kinetics of its clearance was not accelerated compared to wild-type or SpyADrev when normalized to an early time point (Fig. 3I). Combined, these data indicate that SpyAD is important for invasion of pharyngeal epithelial cells *in vitro*.

GAS can cause vulvovaginitis, an understudied but important GAS manifestation generally reported in prepubertal girls (32–34). To examine GAS SpyAD-host cell interactions at an additional site of mucosal colonization, we assessed GAS WT and Δ*spyAD* mutant adherence and invasion to the human vaginal epithelial cell line VK2. In these experiments, the Δ*spyAD* mutant strain showed significant decreases in both adherence and invasion of the VK2 cells (Fig. 3J and K).

M1T1 GAS can produce biofilms, which may interfere with efficient antibiotic eradication (35–37). Using a crystal violet assay for biofilm quantification, we found that loss of SpyAD significantly reduced GAS biofilm formation (Fig. 3L), providing additional *in vitro* evidence that SpyAD may contribute to GAS virulence through multifaceted roles in epithelial cell interactions.

### SpyAD contributes to M1T1 GAS resistance to innate immunity through neutrophil evasion mechanisms

**Fig 4:**
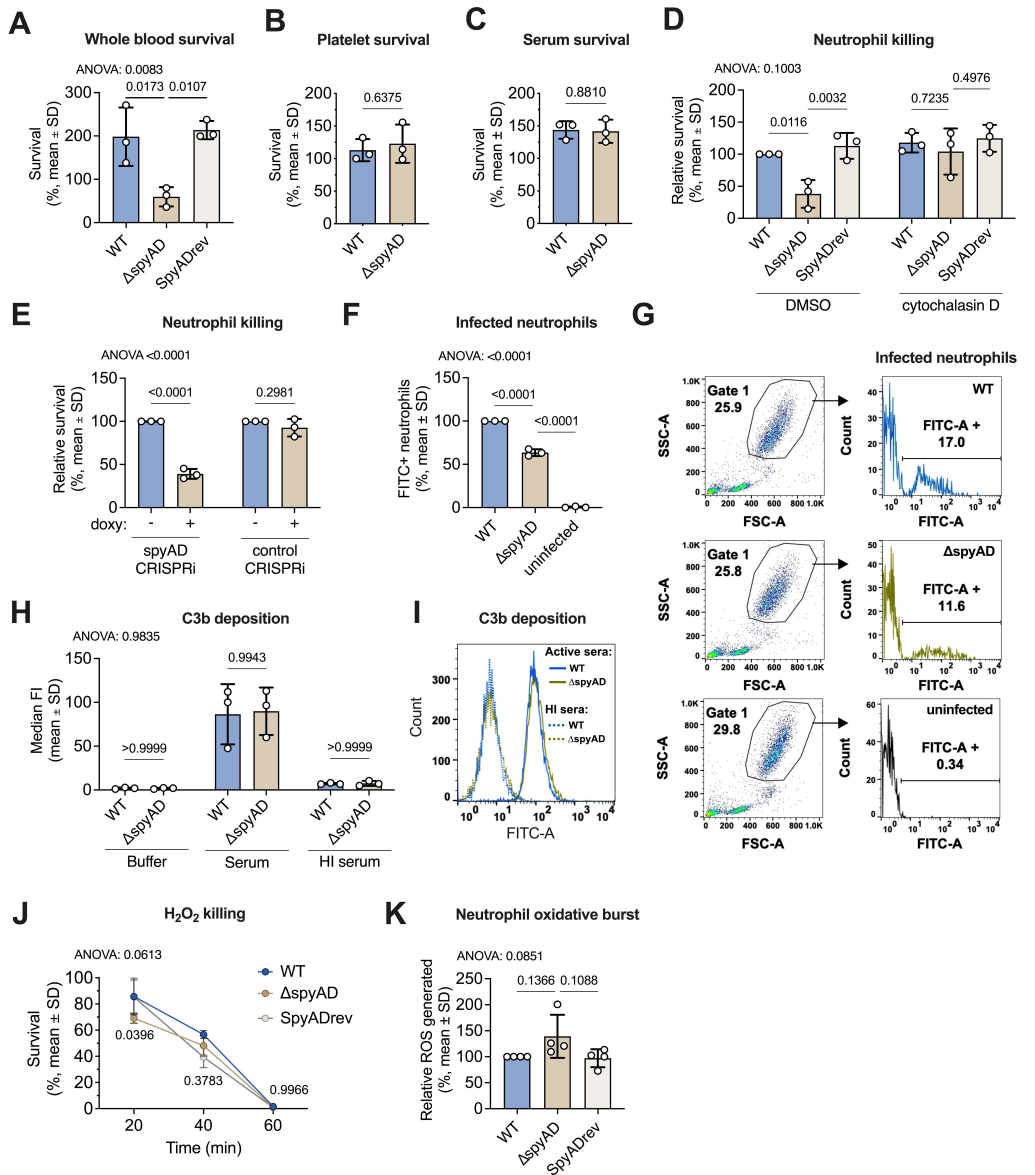
SpyAD contributes to innate immune resistance by M1T1 GAS through neutrophil evasion mechanisms. (**A**) Survival of GAS at a 1:10 dilution of bacteria in whole blood after 1 h of incubation, reported as percent of the initial inoculum. Data show three independent experiments with different blood donors done in triplicate, with each point showing the mean of the three technical replicates. Data are analyzed by one-way ANOVA with Tukey’s multiple posttest (η^2^ = 0.7974). (**B**) Survival of GAS with primary human platelets at MOI = 0.01 for 90 min. Data are recorded as a percent of inoculum and reflect three independent experiments done in triplicate, with each point showing the mean of the three technical replicates. Data are analyzed by Student’s *t* test (η^2^ = 0.06085). (**C**) Bacterial survival at 1 h in 5% pooled human serum. Data are recorded as a percent of inoculum and show three independent experiments done in triplicate, with each point as the mean of the three technical replicates. Data are analyzed by Student’s *t* test (η^2^ = 0.006319). (**D**) Bacteria survival with opsonized primary human neutrophils after 1 h of incubation at MOI = 1. Actin polymerization/phagocytosis inhibitor cytochalasin D was added at 10 µg/mL. Data show three independent experiments, each with a different blood donor, done in triplicate. Each point reflects the mean of the three technical replicates, and each experiment is normalized to the “wild-type +DMSO” condition. Data are analyzed by two-way ANOVA with Tukey’s multiple post-test (interaction η^2^ = 0.1291; ±cytochalasin D factor η^2^ = 0.2229; strain factor η^2^ = 0.3714). (**E**) Bacteria survival with opsonized primary human neutrophils (MOI = 1, 1 h) of SpyAD or control CRISPRi strains grown in the presence or absence of 20 ng/mL doxycycline. Survival is relative to the “minus doxycycline” condition for each strain. Data represent three independent experiments with different donors done in triplicate, and each point is the mean of the three technical replicates. Data were analyzed by two-way ANOVA with Tukey’s multiple post-test (interaction η^2^ = 0.2667; strain factor η^2^ = 0.2667; ±doxy factor η^2^ = 0.4328). (**F**) FITC + fluorescent neutrophils as a percentage of total neutrophils in whole blood cells after 30 min incubation with FITC-labeled wild-type bacteria, ∆*spyAD* bacteria, or no bacteria (uninfected). Data are pooled from three independent experiments done in duplicate. Each point is the mean of the two technical replicates, and each experiment is normalized to blood infected with wild-type GAS. Data are analyzed by one-way ANOVA with Tukey’s multiple post-test (η^2^ = 0.9979). (**G**) Representative flow plot of panel **F** showing gating strategy for neutrophils based on forward and side scatter and gating strategy for FITC+ cells. (**H**) Flow cytometry assay of C3b deposition measured by median fluorescent intensities (median FI) from three independent experiments. Median FI was measured with buffer only, 20% active pooled human serum, or 20% heat-inactivated (HI) pooled human serum. Data were analyzed by two-way ANOVA with Tukey’s multiple post-test (interaction η^2^ = 0.0003356; serum factor η^2^ = 0.8786; strain factor η^2^ = 0.0002025). (**I**) Representative histogram of C3b composition from panel **H**. (**J**) Survival (percent of inoculum) of GAS in 0.05% H_2_O_2_ over time. Data are pooled from three independent experiments done in triplicate and are analyzed by two-way ANOVA with Tukey’s multiple posttest (interaction η^2^ = 0.02092; time factor η^2^ = 0.9332; strain factor η^2^ = 0.01149). (**K**) Oxidative burst capacity of primary human neutrophils with GAS as quantified by dichloro-dihydrofluorescein diacetate fluorescence after 2 h of incubation of neutrophils with bacteria. Data reflect fluorescence units relative to WT-infected neutrophils pooled from four independent experiments with different donors done in triplicate, with each point representing the mean of 3 technical replicates. Data are analyzed by one-way ANOVA with Tukey’s multiple post-test (η^2^ = 0.4216). All graphs represent mean ± SD, and *P*-values are shown above each corresponding comparison. *P*-values of the overall ANOVA test are displayed on the top left corner of the graph when applicable.

We next examined whether SpyAD confers resistance of M1T1 GAS to innate immune responses. The ∆*spyAD* mutant exhibited decreased survival in whole human blood compared to wild type and SpyADrev (Fig. 4A). While no survival differences were observed with primary human platelets (Fig. 4B) or serum (Fig. 4C), the ∆*spyAD* mutant was more susceptible to killing by primary human neutrophils (Fig. 4D). To investigate the role of phagocytosis in this increased susceptibility, we treated neutrophils with the actin polymerization inhibitor cytochalasin D, which eliminated the survival difference between wild type and Δ*spyAD* (Fig. 4D). This suggests that phagocytosis is a key factor in the Δ*spyAD* mutant’s increased susceptibility to neutrophil killing. These findings were further supported by the CRISPRi SpyAD knockdown strain, which also demonstrated impaired survival in neutrophil killing assays (Fig. 4E). Flow cytometry analysis of whole blood infected with FITC-labeled bacteria revealed a lower percentage of infected neutrophils in the Δ*spyAD* mutant group compared to wild type (Fig. 4F and G). However, comparable C3b deposition on wild type and ∆*spyAD* GAS (Fig. 4H and I) suggested that differences in neutrophil killing were not due to increased complement deposition.

To further explore SpyAD’s role in immune evasion, we assessed the ∆*spyAD* mutant’s susceptibility to hydrogen peroxide and neutrophil oxidative burst, a major bactericidal mechanism involving reactive oxygen species (ROS). GAS possesses multiple enzymatic and regulatory mechanisms to suppress and detoxify ROS (38). ∆*spyAD* GAS showed immediate, short-term phase hypersensitivity to killing by H_2_O_2_ (Fig. 4J), but did not significantly alter neutrophil oxidative burst compared to wild-type GAS (Fig. 4K). Overall, SpyAD contributes to M1T1 GAS resistance to killing in whole human blood and primary human neutrophils, possibly through phagocytosis-dependent mechanisms within neutrophils.

### SpyAD protein contributes to M1T1 GAS virulence in systemic infection *in vivo*

**Fig 5:**
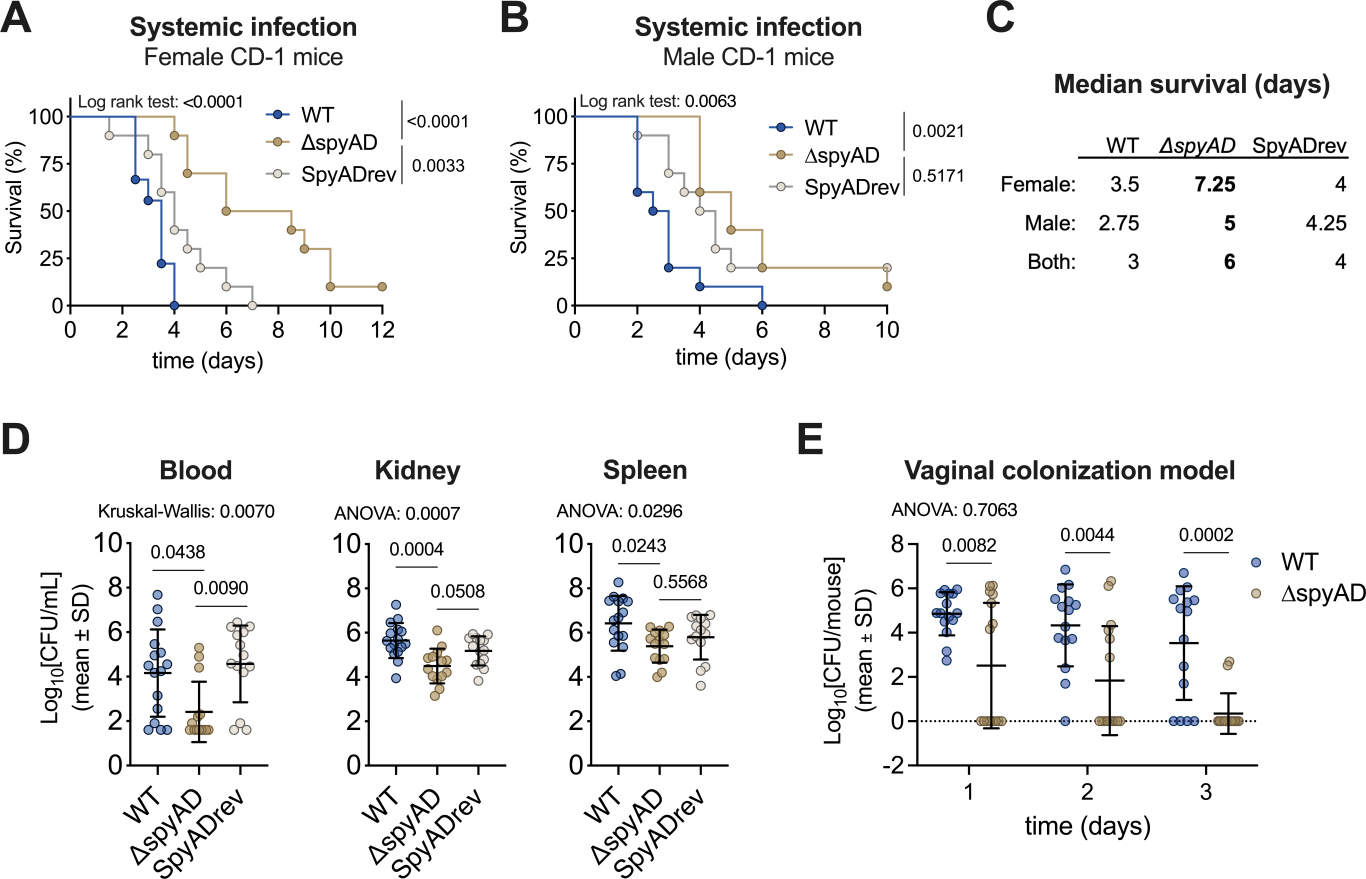
∆*spyAD* shows attenuated virulence in mouse models of M1T1 GAS systemic infection and vaginal colonization. (**A**) Survival rates of female CD-1 mice (*n* = 10/group) after intraperitoneal infection with 2–3 × 10^8^ CFU GAS in 100 µL. Survival is analyzed by log-rank (Mantel-Cox) test. (**B**) Survival rates of male CD-1 mice (*n* = 10/group) after intraperitoneal infection with 3–4 × 10^8^ CFU GAS in 100 µL analyzed by log-rank (Mantel-Cox) test. (**C**) Summary of median survival times from WT-, ∆*spyAD*-, or SpyADrev-infected female mice (from panel A), male mice (from panel B), and combined female and male mice, “both” (A and B). (**D**) Bacterial burden in spleen, blood, and kidney 26 h post-infection with 1–2 × 10^8^ CFU GAS pooled from two independent experiments (WT: *n* = 16, ∆*spyAD*: *n* = 14, and SpyADrev: *n* = 14). Each data point represents one mouse/biological replicate. Bars represent mean ± SD. Kidney and spleen data (middle and right panels) were log-normally distributed (Shapiro-Wilk assessment), and one-way ANOVA with Tukey’s multiple posttest was used to analyze log_10_-transformed data (kidney η^2^ = 0.3008; spleen η^2^ = 0.1577). Blood data (left panel) were not normally or lognormally distributed (Shapiro-Wilk test), so the Kruskal-Wallis non-parametric test was used, followed by Dunn’s multiple comparisons test (η^2^ = 0.193). (**E**) Bacterial burden recovered from vaginal GAS colonization 1–3 days post-infection. Results are pooled from two independent experiments, and each data point shows one mouse/biological replicate. Bars represent mean ± SD. Data were log-normally distributed (Shapiro-Wilk assessment), and two-way ANOVA with Tukey’s multiple posttest was used to analyze log_10_-transformed data (interaction η^2^ = 0.005253; time factor η^2^ = 0.08232; strain factor η^2^ = 0.2804). *P*-values are shown above each corresponding comparison. *P*-values of the overall ANOVA, log-rank, or Kruskal-Wallis test are displayed on the top left corner of the graph when applicable.

Given SpyAD’s role in epithelial invasion and immune evasion, we investigated its contribution to virulence in a murine model of systemic M1T1 GAS infection. Female CD-1 mice injected intraperitoneally with the Δ*spyAD* mutant strain showed significantly prolonged survival (median survival: 7.25 days) compared to mice infected with wild-type (median survival: 3.5 days) and SpyADrev (median survival: 4 days) strains (Fig. 5A). Prolonged survival was also observed with male CD-1 mice infected with ∆*spyAD* GAS compared to wild-type GAS (Fig. 5B). Although most ∆*spyAD*-infected mice ultimately succumbed to infection by the final time point, their median survival time was consistently longer than that of wild-type-infected mice (Fig. 5C). In addition, bacterial burdens in blood, spleen, and kidneys were lower in Δ*spyAD*-infected female mice, particularly in blood, underscoring SpyAD’s role in facilitating systemic infection (Fig. 5D). Given its role in adherence and invasion of vaginal epithelial cells, we lastly evaluated the function of SpyAD in a murine vaginal colonization model. Female mice inoculated intravaginally with the Δ*spyAD* mutant exhibited significantly reduced bacterial loads on days 1–3 compared to those inoculated with wild-type strain (Fig. 5E). These findings suggest that SpyAD is important for initial mucosal colonization and persistence, reinforcing its role in M1T1 GAS survival across multiple infection sites *in vivo*.

## DISCUSSION

Given the ongoing need for effective vaccines against GAS due to its global health impact, understanding GAS virulence factors is crucial. SpyAD, a membrane-bound protein, has garnered attention as a potential vaccine antigen. In this study, we delved deeper into the role of SpyAD in GAS pathogenesis in the M1T1 strain, examining its involvement in epithelial cell interactions, biofilm formation, and evasion of innate immune defenses. We found that SpyAD contributes to both M1T1 GAS systemic infection and vaginal mucosal colonization in mice, supporting its involvement in epithelial cell interactions, resistance to neutrophil killing, and biofilm formation. Key *in vitro* findings, such as SpyAD’s effect on neutrophil killing and epithelial cell invasion, were validated using a CRISPRi-based genetic approach, highlighting the utility of this genetic tool for generating inducible GAS knockdowns. Our findings reveal that SpyAD has multifaceted roles in virulence in the M1T1 GAS strain, with key implications for the prevention and therapy of GAS diseases.

Since the discovery that GAS can invade epithelial cells (39), considerable efforts have been made to elucidate the underlying this process (7, 12, 30, 40, 41). Bacterial invasins like the M protein (42) and fibronectin-binding proteins such as SfbI (43, 44) have been identified as facilitating high-frequency invasion. Our *in vitro* experiments demonstrate that SpyAD is integral to GAS interactions with epithelial cells, particularly in invasion. The loss of SpyAD significantly impaired the ability of M1T1 GAS to invade pharyngeal and vaginal epithelial cells, suggesting that SpyAD contributes to entry into these cell types. This reduction in invasion was not attributable to impaired intracellular survival, as ∆*spyAD* mutant GAS exhibited similar or even enhanced survival within epithelial cells. While the exact mechanism of SpyAD-mediated invasion is not fully understood, SpyAD’s ability to bind human keratin I and collagen VI could mediate its interaction with epithelial cells (19).

Epithelial cell adherence and invasion can be independent events facilitated by distinct surface proteins and integrins (7). Interestingly, our results revealed that SpyAD affects adherence and invasion in a cell-type-specific manner. The ∆*spyAD* mutant was deficient in adhering to vaginal VK2 epithelial cells but not to pharyngeal Detroit 562 cells. The importance of SpyAD in VK2 cell interactions aligns with our *in vivo* findings, which highlighted SpyAD’s role in vaginal mucosal colonization. Future imaging studies of murine vaginal tissue would be interesting to reveal the extent of GAS biofilm formation and epithelial cell invasion in this environment. The ability of GAS to colonize and persist in the vaginal mucosa is relevant to the pathogenesis of vulvovaginitis, and SpyAD may facilitate this colonization, influencing infection persistence in this niche.

GAS has numerous strategies to avoid both extracellular and intracellular destruction by neutrophils, including resistance to oxidative bursts after phagocytosis (14). Our data indicate that SpyAD is important for M1T1 GAS resistance to neutrophil-mediated killing. The Δ*spyAD* mutant strain was significantly more susceptible to opsonophagocytic killing by neutrophils, and this difference was eliminated when phagocytosis was blocked with cytochalasin D. These findings suggest that SpyAD mediates resistance through phagocytosis-dependent mechanisms rather than through extracellular neutrophil-secreted factors. Furthermore, flow cytometry analysis of FITC-labeled whole blood cells revealed that a higher percentage of neutrophils were infected with wild-type bacteria compared to the Δ*spyAD* mutant. Taken together, these data suggest that SpyAD promotes survival within neutrophils, potentially enhancing resistance to intracellular killing.

The importance of SpyAD in innate immune resistance was further corroborated by *in vivo* experiments, where the loss of SpyAD resulted in prolonged survival and reduced bacterial burden in mice with systemic M1T1 GAS infection. The results in this work indicate that SpyAD’s role in GAS virulence likely involves a combination of enhancing epithelial invasion and survival in the bloodstream. Further studies are needed to elucidate the relative contributions of these mechanisms *in vivo*. Our work aligns with a recent study performed in an M28 strain background showing that SpyAD contributes to necrotizing myositis and genital tract infection in non-human primates (NHPs) (18). In these models, the loss of SpyAD resulted in decreased lesion size and CFUs, reinforcing its role in promoting GAS survival within the host (18).

This study investigates the role of SpyAD in a single GAS strain—the predominant and persistent M1T1 lineage. Although M1T1 is frequently isolated from both superficial and invasive infections, the function of SpyAD remains unexplored in other clinically relevant GAS strains, including recently emerged subvariants such as M1_UK_, which has been implicated in invasive infections across the northern hemisphere and Australia (45, 46). While SpyAD is highly conserved across GAS lineages (19), the magnitude of its functional impact may vary among strains due to differences in their virulence factor repertoires. Many GAS virulence factors such as the hyaluronic acid (HA) capsule exhibit differential carriage across GAS strains (47, 48). Thus, even conserved factors such as SpyAD may have varying accessibility or roles depending on the genetic context. Although SpyAD contributes to innate immune resistance in GAS, its relative importance may depend on the presence and expression levels of other virulence factors.

SpyAD has shown promise as a vaccine antigen due to its surface localization, conservation across strains, and ability to elicit opsonizing antibodies (15–17). The protective effects of SpyAD immunization in our murine skin infection model, combined with enhanced opsonophagocytic killing by human blood of GAS opsonized with SpyAD rabbit antisera, underscore its potential as a vaccine target. Targeting SpyAD in a vaccine formulation could reduce the ability of GAS to establish infection, persist within the host, and evade immune responses, providing protection against invasive GAS diseases.

In conclusion, this study provides new insights into SpyAD’s role in the pathogenesis of the predominant M1T1 GAS strain, highlighting its contributions to epithelial cell interactions, biofilm formation, and resistance to innate immune responses. The multifaceted role of SpyAD in M1T1 GAS virulence underscores its potential as a target for therapeutic interventions and vaccine development. Future studies may further investigate the mechanisms by which SpyAD influences GAS pathogenesis across different clinically relevant GAS strains and evaluate its efficacy as a vaccine antigen in preclinical models.

## MATERIALS AND METHODS

### Bacterial strains and genetic manipulations

GAS M1T1 5448 (49) and GAS M1 strain 89155 (50) were used in this study. Insertional mutation of SpyAD in the M1T1 5448 strain was performed with osmotic protection in THB 0.5M sucrose (15, 51, 52). Δ*spyAD* GAS was generated by insertional mutagenesis of pHY304 at the active site, as described previously (15). The “complement” of Δ*spyAD* was made by subcloning the full-length *spyAD* gene into the streptococcal expression vector pDCerm (53). To make the revertant strain SpyADrev, the Δ*spyAD* mutant was passaged in media without antibiotic selection at 37°C, and colonies that lost resistance to erythromycin were selected.

### Bacterial growth conditions

Bacteria were propagated in Todd-Hewitt broth (THB, Hardy Diagnostics) or Todd-Hewitt agar (THA) plates at 37°C in static conditions. Unless indicated otherwise, bacteria were grown to the mid-logarithmic phase for experiments at an optical density at 600 nm (OD_600_) = 0.4. Erythromycin selection was used at 5 µg/mL for Δ*spyAD* GAS for overnight cultures.

### Real-time RT-PCR analysis

RNA was extracted from 10 mL mid-logarithmic phase bacteria using a DNA/RNA Co-Extraction kit (Zymo Research #R2002). Samples were treated twice with DNase using the turbo DNA-free kit (#AM1907). Removal of contaminating DNA was verified by PCR amplification in the absence of reverse transcriptase. Isolated RNA was converted to cDNA using iScript Reverse Transcription Supermix (Bio-Rad #1708840). Gene expression was quantified using an ABI PRISM 7900HT sequence detection system using SpyAD-specific primers 5’GCTTGTTGGCCAAACCTCTG-3′ and 5′-CTGCCAACATGACGACTCCT-3′ in PCR Master Mix (Applied Biosystems #A25742) containing SYBR Green. RNA expression levels of GAS housekeeping gene gyrase A (gyrA) (54) were determined using primers 5′-GAAGTGATCCCTGGACCTGA-3′ and 5′-CCCGACCTGTTTGAGTTGTT-3′. Relative expression amounts were calculated using the delta-delta CT method (55).

### Antimicrobial peptide killing assay

Human cathelicidin LL-37 trifluoroacetate salt (Bachem) was used to treat GAS at 10^6^ CFU/mL in DMEM with 10% THB and 8 µM LL-37. Bacteria were incubated at 37°C for 30 minutes, then CFU were enumerated on THA plates.

### Bacterial growth curve and autolysis analysis

Absorbance at 600 nm was measured every 30 minutes for 5 hours. For growth curve analysis, overnight bacteria cultures were set to OD_600_ of 0.1 in triplicates. For autolysis, log-phase bacteria were washed in ice-cold distilled water and resuspended in PBS containing 0.05% Triton X-100.

### Hyaluronic acid capsule quantification

Five mlliliters of mid-logarithmic phase culture was centrifuged, washed in PBS, and resuspended in 500 µL deionized water. Four hundred milliliters of bacterial suspension was added to 1 mL chloroform, followed by shaking in a Mini-BeadBeater-8 for 5 minutes to disrupt the capsule (56). The aqueous phase was diluted 1:100 in deionized water, and the level of hyaluronic acid was determined using a hyaluronic acid test kit (R&D Systems) according to the manufacturer’s instructions.

### Gram stain and chain length enumeration

Bacteria were gram stained using crystal violet as per manufacturer’s instructions (Hardy Diagnostics #GK400A). Images were taken under 63× objective using an Axio Observer D1 microscope (Zeiss, Göttingen, Germany).

### Sonication to break up chains

Bacterial suspensions were gently sonicated for 2 seconds for three times each on ice for CFU plating analysis to mechanically disrupt chain lengths using a 40TL probe (Ultra Sonic Power).

### Detroit 562 adherence, invasion, and intracellular survival assays

Bacterial association with, and invasion into, Detroit 562 cells was quantified as previously described with minor modifications (57–59). Detroit 562 cells (2 × 10^5^) were seeded in 24-well plates and infected at an MOI of 5:1. Plates were centrifuged and incubated for 30 minutes (adherence) or 2 hours (invasion) at 37°C in 5% CO_2_. At final time points, cells were washed five times with PBS, disrupted with 100 µL of trypsin-EDTA, lysed with 400 µL of 0.025% Triton X-100, and plated on THA for CFU enumeration. For invasion, non-internalized bacteria were killed with 2 hours of incubation in 10 µg/mL penicillin and 100 µg/mL gentamicin, and intracellular bacteria were counted after 2 hours. Intracellular survival was assessed at 4, 6, and 24 hours post-antibiotic addition.

### CRISPRi strain generation

To construct the GAS SpyAD-targeting CRISPRi strain, annealed complementary oligos were ligated to pDC-sgRNA plasmid as described previously for transformation into NV6 GAS strain (31). Briefly, complementary oligos were annealed and phosphorylated using T4 PNK (NEB). Phosphorylated oligos were ligated to digested pDC-sgRNA vector for 1 hour at room temperature. Electrocompetent cells of strain NV6 were transformed with the ligation mixture.

### Flow cytometry of SpyAD surface expression

SpyAD and control CRISPRi strains were grown for 2.5 hours to the mid-logarithmic phase in the presence or absence of 20 ng/mL doxycycline. Bacteria were washed in PBS, set to 10^8^ CFU/mL, and blocked in 10% heat-inactivated donkey serum for 1 hour under rotation at 37°C. SpyAD rabbit antisera (15) was added to a final concentration of 2%, followed by incubation for 1 hour under rotation at 37°C. After washing in PBS, bacteria were resuspended in a 1:200 dilution of Alexa Fluor 488 donkey anti-rabbit IgG (Invitrogen #A21206) for 30 minutes under rotation at 37°C. Samples underwent a final wash, were resuspended in 300 µL PBS, and were run on BD FACS Canto II. Flow cytometry data were analyzed with FlowJo v. 10.8.2 (Tree Star, Inc.).

### Fluorescent microscopy of invasion assay

Detroit 562 cells were seeded at a density of 10^5^ cells per well in 8-well chamber slides (Thermo Fisher #154453). Twenty-four hours later, bacteria were stained with 10× CellBrite Fix stains 488 (Biotium #30090) dye and washed with PBS. The invasion assay was conducted as described previously. The cells were then fixed with 4% paraformaldehyde (PFA) for 15 minutes at room temperature, washed with PBS, and stored in 1% bovine serum albumin (BSA) overnight. Cells were permeabilized with 0.2% Triton X-100 for 10 minutes at room temperature, followed by a PBS wash. The cells were incubated with Alexa Fluor 647-phalloidin (1:40) and DAPI (1:5000) in PBST for 30 minutes, washed twice with PBS, and mounted with Vectashield. Slides were left in the dark for 30 minutes and imaged using an AxioObserver D1 Inverted Fluorescence Microscope (Zeiss).

### VK2 adherence and invasion assays

VK2 vaginal epithelial cells were cultured in 24-well plates in KSFM (Gibco) with rEGF and BPE at 37°C in 5% CO_2_. Bacteria were added at an MOI of 10 and incubated for 30 minutes. Cells were washed, treated with 500 µL of 0.05% Trypsin-EDTA, and 0.25% Triton X-100 for 5 minutes, and bacterial counts were determined by THA plating. For invasion assays, cells were incubated with bacteria for 2 hours, followed by 100 µg/mL gentamicin for 2 hours, then processed as described for CFU enumeration.

### Biofilm quantification

Mid-logarithmic phase bacteria were diluted 1:100 in THB. 200 µL were added to each well of a 96-well plate, followed by incubation for 24 hours at 37°C. Plates were washed three times with PBS and then fixed with 200 µL of 4% PFA for 20 minutes. PFA was then removed, and biofilms were visualized by exposure to 0.2% (wt/vol) crystal violet for 15 minutes at room temperature followed by three PBS washes. The biomass was re-suspended in 200 µL of ethanol/acetone (80:20) for quantification of absorbance at 595 nm.

### Whole blood survival

Human venous blood was drawn from healthy consenting individuals under a protocol approved by the University of California San Diego (UCSD) Human Research Protections Program. 180 µL of heparinized blood was incubated with 2 × 10^4^ CFU of exponential phase bacteria (in 20 µL) in siliconized tubes for 60 minutes at 37°C, followed by CFU enumeration by serial dilution and plating on THA. For assays with immune sera, GAS was incubated for 45 minutes with heat-inactivated rabbit sera from SpyAD-immunized rabbits (15) immediately prior to the addition of whole blood for a final rabbit serum concentration of 8%.

### Platelet isolation and killing assay

Human platelets were isolated from fresh blood obtained from healthy donors using acid-citrate-dextrose buffer (ACD; 1:6 [vol/vol]; Sigma Aldrich) as an anticoagulant following an established method (60). To evaluate the bacterial killing by platelets, isolated platelets were infected at MOI = 0.01 (1 × 10^7^ platelets with 1 × 10^5^ CFU of the bacteria) for 90 minutes at 37°C in 5% CO_2_. After incubation, infected platelets were sonicated (Fisher Sonic Dismembrator 550) 2 times for 3 seconds, diluted, and plated on THA plates.

### Serum survival

Pooled serum from six healthy human donors was added to 1 mL RPMI in siliconized tubes for a final concentration of 5% serum and 1 × 10^6^ CFU/mL GAS. Bacteria were incubated in serum at 37°C for 1 h under rotation. Following incubation, bacterial counts were assessed by plating on THA.

### Neutrophil isolation and killing assay

Human whole blood and serum were collected from healthy donors. Neutrophils were isolated from fresh human blood by density gradient centrifugation using Polymorphprep (Alere Technologies). Immediately prior to the assay, bacteria were incubated for 30 minutes with pooled human serum for opsonization. Exponential phase bacteria (2 × 10^5^ CFU/100 µL) were mixed with human neutrophils (2 × 10^5^ cells/100 µL) in Hanks’ Balanced Salt Solution (HBSS, Thermo Fisher) for an MOI of 1 with 5% pooled human serum. The 96-well plate was then incubated at 37°C with 5% CO_2_ for 1 hour. Cell counts were assessed by plating on THA. To inhibit actin polymerization and phagocytosis, neutrophils were incubated for 30 minutes in cytochalasin D prior to the assay to a final concentration of 10 µg/mL.

### Flow cytometry of infected whole blood

Infection and flow cytometry of whole blood were done as described previously, with some modifications (61). Bacteria were suspended in PBS with 0.5 mg/mL fluorescein isothiocyanate (FITC; Sigma-Aldrich) for 20 min under shaking conditions and protected from light. After three washes in PBS, bacteria were then resuspended in RPMI to an OD_600_ = 0.4. Forty microliters of FITC-labeled GAS was then added to 160 µL of heparinized freshly isolated human blood in FACS tubes for 30 min at 37°C with 5% CO_2_ under shaking conditions. The phagocytosis reaction was stopped with 2 mL of fluorescence-activated cell sorter lysing solution (BD Biosciences) for 10 min at room temperature. Samples were washed two times in RPMI, and 10,000 cells were analyzed using flow cytometry (BD FACS Canto II, FlowJo v10.8.2). The gating of cells was based on forward and side scatter. Internalized bacteria were identified when neutrophils expressed fluorescence over uninfected controls.

### Complement (C3b) deposition

Bacteria (10^6^ CFU/mL) were incubated with 20% pooled human serum in RPMI-0.1% BSA (200 µL) for 45 minutes at 37°C under rotation. After washing twice in RPMI-0.1% BSA, bacteria were incubated with 1 µg/mL anti-C3b antibody (Sigma) for 1 hour at 37°C, followed by Alexa Fluor 488-conjugated secondary antibody for 30 minutes under the same conditions, with two washes in between. After a final wash, samples were resuspended in PBS, transferred to FACS tubes, and analyzed using flow cytometry (BD FACS Canto II, FlowJo v10.8.2).

### Hydrogen peroxide sensitivity assay

Bacteria at OD_600_ = 0.4 and incubated in 100 µL 0.05% H_2_O_2_ (Thermo Fischer) in THB at 37°C. Survival of GAS in 0.05% H_2_O_2_ was assessed at 20, 40, and 60 minutes by CFU enumeration through plating on THA. Catalase was added to the first row of dilution plates at 1 mg/mL to quench residual H_2_O_2_.

### Neutrophil oxidative burst

Freshly isolated neutrophils from healthy human donors were incubated in 2.5 mM dichloro-dihydrofluorescein diacetate (DCFH-DA) at 2 × 10^6^ cells/mL in HBSS under rotation for 20 minutes at 37°C. Neutrophils (final of 2 × 10^5^ neutrophils per well) were added to a 96-well tissue culture plate with bacteria at MOI = 10 or 25 nM PMA in HBSS medium supplemented with calcium and magnesium. The plate was centrifuged for 500 × *g* for 5 minutes to ensure contact with bacteria and neutrophils and incubated at 37°C with 5% CO_2_. Fluorescence intensity was measured at 485 nm excitation/520 nm emission after 2 h.

### Murine studies

Mice were kept on a 12 hour light/dark schedule, fed a 2,020× diet (Envigo), and provided acidified water. Before experimentation, mice were randomly assigned to cages with no more than 5 mice per cage. The mouse experiments were approved by the UC San Diego Institutional Animal Care and Use Committee (Protocol #S00227M) in compliance with federal regulations and were conducted under the oversight of veterinary professionals.

### Murine immunizations and intradermal challenge

Wild-type female CD-1 mice (Charles River) were immunized with recombinant SpyAD every 14 days for three doses, starting at 5–7 weeks of age. Each intramuscular immunization (100 µL) included 50 µL of Alhydrogel 2% aluminum hydroxide adjuvant (Invivogen). For the intradermal challenge, mice were shaved, depilated, and infected with 10^6^ CFU of M1 89155. Lesion development was monitored daily, and on day 3, mice were euthanized, lesions excised, homogenized, and plated on tryptic soy agar with 5% sheep’s blood for CFU counts. Lesion sizes were measured using FIJI (62).

### Murine systemic infections

Eight-week-old, CD-1 mice (Charles River Laboratories) were intraperitoneally infected with wild-type, ∆*spyAD*, or SpyADrev. For survival curve analysis, 2–3 × 10^8^ CFU for female mice and 3–4 × 10^8^ CFU for male mice in 100 µL of each strain (*n* = 10/group) were intraperitoneally injected, followed by survival assessment twice daily for 12 days (female mice) or 10 days (male mice). For organ CFU enumeration, female mice were infected with 1–2 × 10^8^ CFU in 100 µL of each strain (*n* = 14–16/group). To determine bacterial load, mice were euthanized humanely 26 hours post-infection using CO_2_ followed by cervical dislocation, in accordance with approved IACUC protocols. Blood (obtained by cardiac puncture), kidneys, and spleens were collected. Kidneys and spleens were homogenized using a bead-beater, serially diluted in PBS, and plated on THA.

### Mouse model of vaginal colonization

Female CD-1 mice aged 6–8 weeks were used for all experiments. Experiments were performed as previously described (63, 64). Briefly, 1 day before inoculation, mice received an intraperitoneal injection of 0.5 mg β-estradiol valerate in 100 µL sesame oil to synchronize estrus. On day 0, mice were vaginally inoculated with 10^9^ CFU/mL bacteria in 10 µL PBS. Vaginal lavage was performed on days 1, 2, and 3 with 50 µL sterile PBS, collected, and processed for CFU counts by plating on CHROMagar StrepB.

### Statistical analysis

Statistical analysis was performed using GraphPad Prism 10.2. Each data set was analyzed by the Shapiro-Wilk normality and/or lognormality test. Data that were normally or lognormally distributed were analyzed by two-tailed Student’s *t*-test when comparing two conditions, one-way ANOVA with Tukey’s post-test for data with multiple conditions, or two-way ANOVA with Tukey’s post-test for data with multiple independent variables. Data that were not normally distributed were analyzed by the Kruskal-Wallis test followed by Dunn’s test for multiple comparisons. Mortality curves were compared using the log-rank (Mantel-Cox) test. *P*-values are indicated on each graph, and, in general, *P*  <  0.05 was considered significant. Flow cytometry was analyzed with FlowJo v10.8.1.
